# Effects of Total Dissolved Gas Supersaturation in Fish of Different Sizes and Species

**DOI:** 10.3390/ijerph16132444

**Published:** 2019-07-09

**Authors:** Shudan Xue, Yuanming Wang, Ruifeng Liang, Kefeng Li, Ran Li

**Affiliations:** State Key Laboratory of Hydraulics and Mountain River Engineering, Sichuan University, Chengdu 610065, China

**Keywords:** total dissolved gas supersaturation, upper Yangtze river, endemic fish, species, size, catalase

## Abstract

Two endemic fish in the upper Yangtze River, the Rock Carp (*Procypris rabaudi*) and Prenant’s Schizothoracin (*Schizothorax prenanti*), were used as research objects in this study to assess the effects of total dissolved gas (TDG) supersaturation on fish of varying sizes. Fish were exposed to TDG-supersaturated water at the levels of 145, 140, 135, 130, and 125%. The results showed that fish swam slowly, responded clumsily, and then exhibited spiral swimming performance after a period of exposure to TDG-supersaturated water. Fish exhibited exophthalmos, body swelling, gill bleeding, and caudal fin bleeding when they died in the TDG-supersaturated water. With the increase in TDG supersaturation, the tolerance capacity of fish to supersaturated TDG significantly reduced. At high supersaturation, the difference in survival time between species was not significant, while fish with smaller sizes showed greater tolerance capacity. At low supersaturation, the tolerance capacity of fish was mainly affected by species, and the influence of size was relatively small. With the decrease in TDG supersaturation, the catalase (CAT) activity first increased and then decreased. Rock Carp displayed significantly less activity than Prenant’s Schizothoracin on exposure to TDG-supersaturated water. At high supersaturation levels, the CAT activity of Prenant’s Schizothoracin of small size was greater than that of large Prenant’s Schizothoracin. In contrast, small Prenant’s Schizothoracin showed less CAT activity at low TDG levels than did large individuals.

## 1. Introduction

Total dissolved gas (TDG) supersaturation is a physical condition in which the pressures of atmospheric gases in a solution exceed the barometric pressure [1], and this condition often occurs downstream of dams during the flood season [2,3,4] Large amounts of gases are taken into the downstream energy dissipation pool by high-speed turbulent water. The excess dissolved gas cannot be completely released within a short time due to the hydrostatic and hydrodynamic pressure in the energy dissipation pool, thereby resulting in TDG supersaturation in the downstream river water [5,6,7]. The influence of TDG supersaturation on river ecosystems has become an ecological environmental issue of global concern [8,9,10]. Endemic fish in the Columbia River of the United States suffered mortality caused by TDG supersaturation when hydropower structures discharged flows during the flood season [11,12,13]. Similar phenomena were also found downstream of the Danube River [14]. In recent years, a large number of hydropower projects with a high head (i.e., over 200 m or even as high as 300 m) have been planned or built in the upper Yangtze River [15,16]. Field observations have found that the TDG supersaturation downstream of these hydropower projects ranged from 116.0% (Dachaoshan Dam) to 142.5% (Gongzui Dam) [5], which led to large-scale fish mortality and prompted serious concern in China [17]. Although the restoration of the ecological environment of the Yangtze River has been emphasized since 2018 [18], hydropower development in the upper Yangtze River seems to be continuing, and TDG supersaturation will remain the primary issue that needs to be solved in the near future.

Numerous studies have shown that supersaturated TDG always deteriorates the growth, swimming ability, and hatching of fish [19,20,21,22], as well as predation capacity [23]. TDG supersaturation even causes fish mortality when the supersaturation exceeds a certain level. The tolerance threshold of fish to TDG supersaturation is a vital reference index for the management of flood discharge in hydropower projects. To protect endemic fish that live downstream from dams, the United States has promulgated a TDG criterion of 110% in rivers [24]. For endemic fish in China, especially those inhabiting the upper Yangtze River, the existing research has been relatively simple, and has focused on exploring the response of specific fish to TDG supersaturation [25,26,27,28]. To date, no studies have systematically assessed the impact of fish species and size on tolerance to TDG supersaturation.

The upper Yangtze River of China has abundant fish resources. The State Council of China approved the Rare and Endemic Fish National Nature Reserve in the upper Yangtze River in April 2000 to protect the rare and endangered fish that inhabit the upper Yangtze River [29,30]. Three endangered species and 65 endemic species were listed as objects of protection. In this paper, two species of different sizes in the upper Yangtze River were selected to explore the effects of species and size on fish tolerance characteristics to TDG supersaturation.

In addition, through a long period of evolution, organisms have formed antioxidant defense systems to prevent damage from toxic substances [31]. As organisms have been subjected to environmental stress, the antioxidant defense system changed to protect the internal tissues of their body from attack [32]. Catalase (CAT) in the antioxidant defense system was selected as an index in this paper and was used to evaluate the response of fish to TDG supersaturation. CAT activities were determined at different TDG supersaturation levels, and the differences shown in the different species and sizes were analyzed. The results of this paper can provide a basis for determining the tolerance threshold of endemic fish in the upper Yangtze River to TDG supersaturation.

## 2. Materials and Methods

### 2.1. Experimental Fish

Rock Carp (*Procypris rabaudi*) and Prenant’s Schizothoracin (*Schizothorax prenanti*) are the main fish of economic value that inhabit the upper Yangtze River, and belong to benthic species [33]. The population of these species decreased sharply with hydropower construction. They have reached the third and the second levels of urgent protection, respectively [34].

Healthy fish from the Sichuan Fisheries Research Institute of China were used in the experiments. Before the experiment, fish were held in a tank with saturated equilibrium water (100% TDG, 7.5–8.0 mg/L of dissolved oxygen, water temperature of 20–22 °C, and pH of 7.1–7.8) for one week. Fish were fed earthworms once per day during domestication, and fasted for 24 h before the experiment to eliminate the effect of food. Fish were divided into four groups to conduct the following experiments. Detailed information about the fish is shown in Table 1.

### 2.2. Acute Lethality Experiment 

TDG-supersaturated water was generated by a circulating system that was described in detail by Wang et al. [26]. Experimental fish were held in five plastic tanks (65 cm × 40 cm × 30 cm) with TDG-supersaturated water. The TDG levels were set as 145, 140, 135, 130, and 125%. A control level of 100% TDG was also simultaneously used. Fifteen fish were placed in each tank and TDG supersaturation levels were measured using a Point 4 Tracker (Point Four Systems, Coquitlam, BC, Canada). Fish were immediately removed from tanks once they were identified as dead. The time of death of the individual was recorded, as was the length and weight. An individual was regarded as dead when it had no stress response within 5 min to belly touching by a thin glass rod [35]. Any abnormal behaviors were also recorded during the exposure process.

### 2.3. Measurement of CAT Activity 

When a dead fish was removed from the acute lethality experiment, its muscle tissue (0.15 g) was immediately collected and then lapped with freezing liquid nitrogen. Fresh phosphate buffer solution with a volume of 1.5 mL was simultaneously added to extract the enzyme solution. The extract enzyme was centrifuged for 3 min at 4 °C and 10,000 r/min, and the supernatant was used to determine CAT activity using an ultraviolet spectrophotometer. Three milliliters of reaction mixture (0.1 mL of enzyme extract solution, 2.75 mL of phosphate buffer solution, and 0.15 mL of 0.6% hydrogen peroxide solution) were used to determine the absorbance at a wavelength of 240 nm, and the CAT activity was calculated according to the absorbance.

### 2.4. Statistical Analysis 

In this paper, the survival rate and average survival time were calculated to reflect the tolerance characteristics of fish to supersaturated TDG. The median lethal time (LT_50_) refers to the exposure time at which the mortality of the test fish reached 50% under a specific TDG supersaturation level. The LT_50_ was used to assess the lethality of TDG-supersaturated water to experimental fish. The graphical methods of Miller and Sunter were used to calculate the LT_50_ at different supersaturation levels [36].

CAT activity was used to evaluate the biological response of fish to TDG-supersaturated water. The activity unit of CAT was defined as the amount of the enzyme that caused a decrease of 0.01 on the absorbance of H_2_O_2_ per min under the wave length of 240 nm, which is expressed as follows:

(1) $$CATactivity=\frac{\Delta A\times V_{1}}{0.01\times V_{2}\times m}$$

where *ΔA* is the absorbance difference value after a 1.5-min measurement; *V*_1_ is the total volume of sample liquid, mL; *t* is the measurement time, 1.5 min; *V*_2_ is the volume of sample liquid for measurement, mL; and *m* is the sample mass, g.

The effects of species and size on fish tolerance characteristics were determined using one-way analysis of variance (ANOVA), which was followed by a post hoc multiple comparison test (least significant difference) to determine the difference between the values of the different treatment groups. Tamhane’s T2 test was used when there was inhomogeneous variance. The level of significant difference was set to *p* < 0.05.

## 3. Results

### 3.1. Acute Lethal Symptoms 

**Figure 1:**
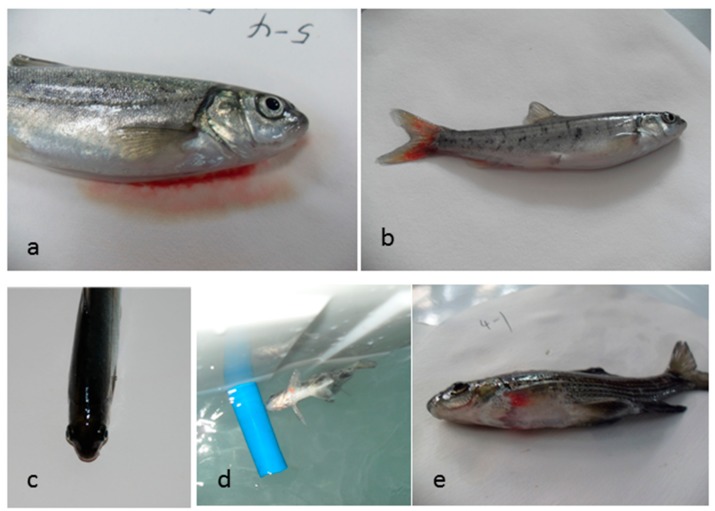
Typical signs of the experimental fish after removal from the total dissolved gas (TDG)-supersaturated water, including (**a**) gill bleeding, (**b**) caudal fin bleeding, (**c**) exophthalmos, (**d**) spiral swimming, and (**e**) body swelling.

The fish in the TDG-supersaturated water swam normally during the first hour of exposure. Ninety minutes later, the fish swam rapidly and jumped up and down at the TDG supersaturation levels of 145, 140, and 135%. After 2 h of exposure, the fish responded clumsily and swam slowly. Spiral swimming performance was found in some fish, and then they gradually lost swimming capacity and remained motionless at the bottom of the tank, finally suffering death. Similar abnormal behavior also existed in the fish at the TDG supersaturation levels of 125 and 130%, although the behavior started a little later than that in fish at the levels of 145, 140, and 135%. The experimental fish exhibited signs of exophthalmos, body swelling, gill bleeding, and caudal fin bleeding when they died (Figure 1). The scales of some fish were found to be falling off their bodies during the exposure process. No significant difference was found in signs among the four groups.

### 3.2. Acute Lethality Experiment 

**Figure 2:**
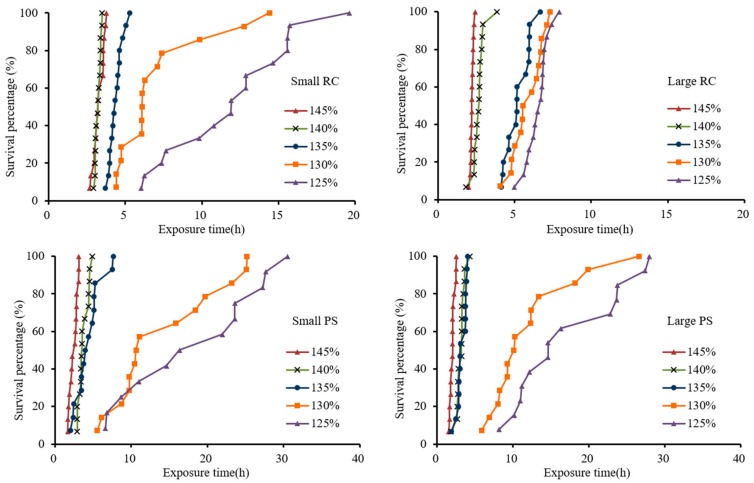
The survival of fish varied with the exposure time at the different TDG supersaturation levels; RC: Rock Carp; PS: Prenant’s Schizothoracin.

The survival time of the experimental fish in each group was shorter and more concentrated with increasing TDG supersaturation. For Rock Carp (RC) in the small RC group, the first fish died at between 2.7 and 6.1 h in the TDG supersaturation levels of 125, 130, 135, 140 and 145%. As the level of supersaturation increased, the fish died faster. At high supersaturation (135, 140, and 145%), 100% death occurred within 5.3, 3.5, and 3.8 h, respectively, while the time exceeded 14 h at low supersaturation (125 and 130%, respectively) (Figure 2a).

The survival time of Rock Carp in the large RC group was shorter at the TDG supersaturation levels of 140 and 145% than in the small RC group. The death times of the first fish in the large RC group were 1.9 and 2.0 h at the TDG supersaturation levels of 140 and 145%, respectively, and the times to 100% mortality were 3.9 and 2.4 h, respectively. The experimental fish at the level of 135% survived longer than did those at 140 and 145%. At the level of 135%, the first fish died at 4.1 h. At the TDG supersaturation level of 135%, there was no significant difference compared with the Rock Carp in the small RC group. Unlike the Rock Carp in the small RC group, the survival of Rock Carp in the large RC group was more concentrated at the levels of 125 and 130% (Figure 2b).

The survial pattern of Prenant’s Schizothoracin was similar to that of Rock Carp. The survival time of Prenant’s Schizothoracin was more concentrated at the supersaturation levels of 130, 140, and 145% than at the levels of 125 and 130%. The mortality rate reached 100% within 7.6 h at the levels of 135 to 145%, and Prenant’s Schizothoracin (PS) in the small PS group reached 100% mortality earlier than the fish in the large PS group. At the TDG supersaturation levels of 125 and 130%, Prenant’s Schizothoracin in the small PS group died between 5.6 and 30.5 h, while fish in the large PS group died between 5.9 and 28.7 h, which was significantly longer than the time to mortality for Rock Carp (Figure 2c,d).

**Figure 3:**
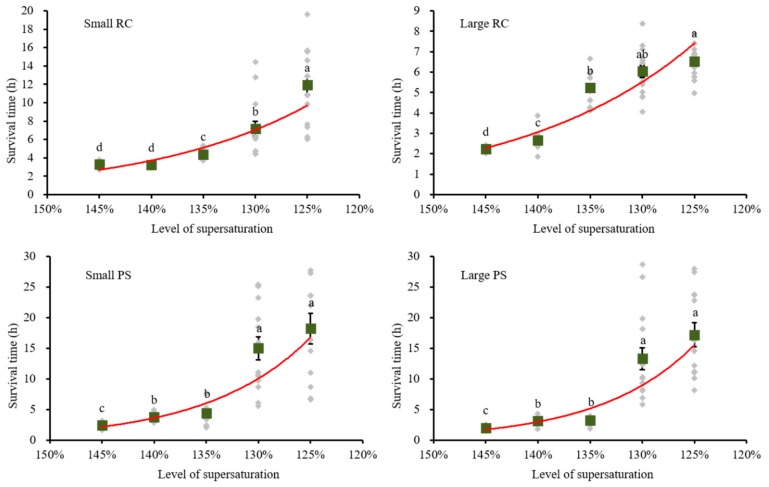
Relationship between survival time and level of supersaturation. Green dots indicate the average survival time. Values are expressed as the mean ± SE, *n* = 12–15. Gray dots indicate the survival time of the individual. Red lines indicate the best-fit regression models. Letters above the dots indicate the results from a post hoc multiple comparison test between different supersaturation level. Mean values that do not share a common lowercase letter are significantly different (*p* < 0.05).

The average survival times of Rock Carp in the small RC group were 3.28 and 3.23 h at the 145 and 140% supersaturation levels, respectively. No significant difference was found between the two levels in terms of time of death. The average values were 4.4, 7.2, and 11.9 h at the levels of 135, 130, and 125%, respectively, increasing significantly with the decrease in TDG supersaturation (*F* = 39.90; *df* = 4, 69; *p* < 0.001; Figure 3a).

The average survival times of Rock Carp in the large RC group were 2.2 and 2.7 h under TDG levels of 145 and 140%, respectively. Similar to the fish in the small RC group, the average survival time of fish in the large RC group increased significantly with the decrease in the TDG supersaturation (*F* = 108.39; *df* = 4, 70; *p* < 0.001; Figure 3b). At the TDG supersaturation levels of 135, 130, and 125%, the average survival times were 5.2, 6.0, and 6.5 h, respectively.

The survival pattern of the small PS group was consistent with that observed in the large PS group. One-way ANOVA indicated that the survival time of fish significantly increased with the decrease in TDG supersaturation (for the small PS group: *F* = 29.79, *df* = 4, 66, *p* < 0.001; and for the large PS group: *F* = 36.39, *df* = 4, 68, *p* < 0.001; Figure 3c,d). Post hoc multiple comparisons showed that the survival time at the levels of 140 and 135% was not significantly different, nor were the survival times at levels of 130 and 125% significantly different.

At high supersaturation (145, 140, and 135%), as shown in Table 2, the difference in the average survival time between species was not significant. Fish with large lengths and weights died more quickly at the TDG supersaturation levels of 145, 140, and 135%, while the difference was significant only at 145% TDG (*F* = 35.38; *df* = 3, 57; *p* < 0.001).

Species was the main factor affecting the tolerance capacity of fish at low TDG supersaturation (125 and 130%) (Table 2). The average survival times of Prenant’s Schizothoracin in the small PS group and in the large PS group were greater than those of Rock Carp (ANOVA for 130%: *F* = 9.47; *df* = 3, 56; *p* < 0.001; ANOVA for 125%: *F* = 13.44; *df* = 3, 52; *p* < 0.001). However, the effect of size on tolerance capacity was not obvious at 125 and 130% TDG supersaturation. There was no significant difference between Prenant’s Schizothoracin in the two groups under the levels of 125 and 130%. Rock Carp in the two groups also did not show a significant difference at the TDG supersaturation level of 130%. The average survival time of Rock Carp in the small RC group was 5.4 h longer than that in the large RC group under the TDG supersaturation level of 125%.

**Figure 4:**
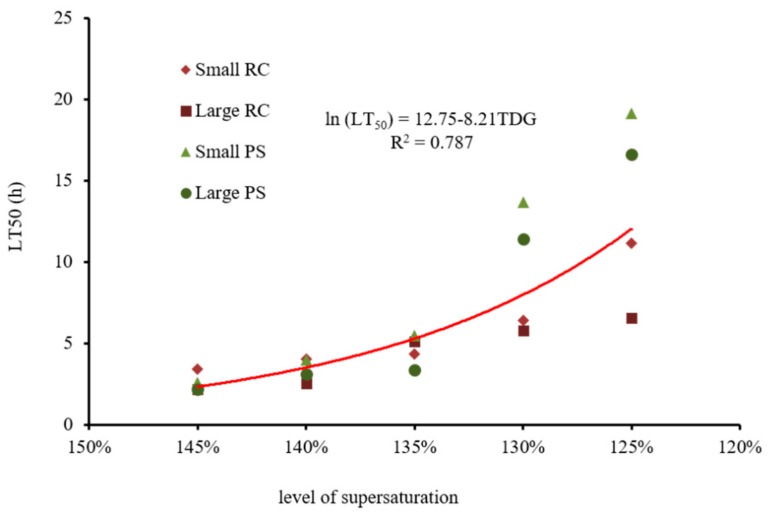
Changes in the median lethal time (LT_50_) of experimental fish with different TDG supersaturation levels.

At a TDG supersaturation level of 145%, the LT_50_ values of Rock Carp in the small RC and the large RC groups and Prenant’s Schizothoracin in the small PS and large PS groups were 2.61, 2.18, 3.40, and 2.19 h, respectively. The LT_50_ gradually increased with the decrease in TDG supersaturation, and increased to 19.14, 16.59, 11.14, and 6.54 h, respectively, at 125% TDG. There was a negative exponential correlation between the LT_50_ and TDG supersaturation level (ln (LT_50_) = 12.75−8.21 TDG, *R^2^* = 0.787) (Figure 4).

### 3.3. CAT Activity 

There was no significant difference in CAT activity for Rock Carp in the large RC group at different TDG supersaturation levels (*F* = 0.97; *df* = 4, 36; *p* = 0.437). Although the CAT activity of Prenant’s Schizothoracin in the large PS group changed greatly, with a range of 82.05−116.89 U/g, ANOVA showed that there was no significant difference in CAT activity at different TDG levels (*F* = 2.41, *df* = 4, 62, *p* = 0.059). For Prenant’s Schizothoracin in the small PS group, the CAT enzyme activity showed no significant difference at high supersaturation levels (145, 140, and 135%), and CAT enzyme activity at 130% was significantly higher than at 145, 140, and 135% (*F* = 3.38; *df* = 4, 51; *p* = 0.016).

As shown in Table 3, the results of one-way ANOVA indicated that there were significant differences in CAT activity among fish species at different TDG levels. The average CAT activity of Rock Carp in the large RC group was significantly lower than that of Prenant’s Schizothoracin at each TDG level. At high supersaturation levels (145 and 140%), the average CAT activity of Prenant’s Schizothoracin in the small PS group was higher than that of Prenant’s Schizothoracin in the large PS group. In contrast, Prenant’s Schizothoracin in the small PS group had a lower CAT activity at the TDG levels of 135, 130 and 125% compared with fish in the large PS group.

The average CAT activity at 130% TDG for all test fish was higher than that of the control group, indicating that the CAT activity was stimulated. In contrast, at the high TDG levels of 135–145%, most test fish had lower CAT activity relative to control fish, except for Prenant’s Schizothoracin in the small PS group at 140% TDG. This result illustrated that the CAT activity of fish was inhibited at higher supersaturation levels. However, it must be noted that there was no significant difference in the mean value of enzyme activity at each supersaturation level (Table 4).

## 4. Discussion

### 4.1. Acute Lethality Experiment 

In this study, at their time of death, the four groups of test fish exhibited exophthalmos, body swelling, gill bleeding, and caudal fin bleeding in supersaturated TDG water, which is in agreement with the research of Wang et al. [26]. During the exposure to TDG supersaturation, the scales of some fish were found to be falling off their bodies. This condition may be caused by furious movement and frequent collisions with the walls of the experimental tanks. No significant difference was found in signs among the four groups, which meant species and size had little effect on the symptoms of gas bubble disease.

At high TDG supersaturation, the test fish in the four groups died rapidly. The death was relatively concentrated, and the TDG supersaturation was the main reason for death. The survival time changed greatly when the supersaturation level was below 135%. We speculate that size and species differences significantly contributed to our results.

Previous studies have shown that different fish species exhibit different tolerance capacities to TDG supersaturation. By comparing the LT_50_ of five different fish species dwelling in the Columbia River Basin, Beeman et al. concluded that the tolerance of different fish to TDG-supersaturated water was different under the same test conditions [37]. In this paper, species had a greater impact on fish tolerance at 130 and 125% TDG supersaturation. The tolerance capacity of Rock Carp was significantly weaker than that of Prenant’s Schizothoracin. The LT_50_ of Rock Carp was only 48.6 and 49.5% of that of Prenant’s Schizothoracin at 130 and 125% TDG supersaturation, respectively.

Size also played an important role in affecting fish tolerance to TDG supersaturation. Robert and Rucker found that the LT50 of the smaller salmon (approximately 38 mm and 46 mm) was approximately 10 times that of the larger salmon (approximately 100 mm) [38]. The mortality of salmon with a body length of approximately 100 mm was approximately 3/4 of that with a body length of 40 mm [39]. In this paper, both Rock Carp and Prenant’s Schizothoracin of larger size had lower LT_50_ than did those of smaller size.

### 4.2. CAT Activity 

With the decrease in TDG supersaturation, the CAT activity first increased and then decreased. The maximal value was reached at 130% TDG, which is in agreement with the law of Chen et al. [31]. At each supersaturation level, the CAT activity of Rock Carp was lower than that of Prenant’s Schizothoracin, which indicated that Prenant’s Schizothoracin had a stronger ability to decompose excessive H_2_O_2_ than did Rock Carp. Such a result was consistent with the result of LT_50_, which both indicated that the tolerance capacity of Rock Carp was weaker than that of Rock Carp. For Prenant’s Schizothoracin, the CAT activity was higher in fish of larger size at high supersaturation levels (145 and 140%). In contrast, Prenant’s Schizothoracin of larger size had lower activity at TDG levels of 135, 130 and 125%.

In this study, the CAT activity of fish was inhibited at high supersaturation. The CAT activity reached a maximum at 130%, and it decreased at a TDG supersaturation level of 125%. It is possible that, at high TDG supersaturation, the toxicity of TDG-supersaturated water seriously damaged the bodies of exposed fish. The system of CAT production in fish was damaged, affecting the ability of fish to decompose excess H_2_O_2_. Therefore, the CAT activity of the test fish was lower than that of the control group. At 125%, the external stimulation completely activated the CAT production system. At the TDG supersaturation level of 130%, fish can produce CAT enzymes to resist the toxic effects of TDG supersaturation and decompose excess H_2_O_2_.

### 4.3. Suggestions

This study considered two fish species, and the fish used from both species were juveniles. Considering that they are susceptible to TDG supersaturation, it is important to research their tolerance characteristics to TDG supersaturation. However, such works are not sufficient, and more research should be conducted in the future.

In recent years, an increasing number of high dams have been built and operated in China [15,16]. TDG supersaturation is becoming increasingly serious [8,10,13]. At present, the amount of research on endemic fish affected by TDG supersaturation in the upper Yangtze River is not rich or sufficient. The evaluation system of the impact of TDG supersaturation on fish needs to be established, and the research work on tolerance to TDG supersaturation needs to be systematic and more profound. The solubility of gases increases with water depth, leading to a decrease in TDG supersaturation. Fish can swim deeper to avoid the threat of TDG supersaturation in natural rivers. When setting the TDG criterion of the upper Yangtze River in the foreseeable future, factors such as species, body shape, life stage, compensation depth, and water temperature should be considered comprehensively [3,25,26,40,41]. 

## 5. Conclusions

Through the study of the tolerance characteristics of Rock Carp and Prenant’s Schizothoracin of different sizes to TDG supersaturation in laboratory, the following conclusions can be drawn:

1. After a period of exposure to TDG-supersaturated water, fish swam slowly, responded clumsily, and then started spiral swimming. Fish exhibited exophthalmos, body swelling, gill bleeding, and caudal fin bleeding when they died in the TDG-supersaturated water. Species and size had little effect on the symptoms of bubble disease.
2. At high TDG levels (135% and above), the difference in average survival time between different species was not significant, and fish of smaller size showed greater tolerance capacity. At low TDG levels (125 and 130%), the tolerance capacity of fish to supersaturated TDG was mainly affected by species, and the influence of size was limited.
3. With the decrease in the TDG supersaturation, the CAT activity first increased and then decreased, and reached a maximum value at 130% TDG supersaturation. The CAT activity was stimulated at levels of 125–130%, and was inhibited at 135% TDG and above. At different supersaturation levels, the average CAT activity of Rock Carp was significantly lower than that of Prenant’s Schizothoracin. At levels of 140 and 145%, the average CAT activity of Prenant’s Schizothoracin of small size was greater than that of large Prenant’s Schizothoracin. In contrast, small Prenant’s Schizothoracin had lower activity than did the large individuals at levels of 125–135%.

## Figures and Tables

**Table 1:** Length and weight of experimental fish. Values are expressed as the mean ± SE. Letters following the values indicate the results from a post hoc multiple comparison test between groups. Mean values that do not share a common lowercase letter are significantly different Means not sharing a common lowercase letter are significantly different (ANOVA for length: *F* = 20.70; *df* = 3, 289; *p* < 0.001. ANOVA for weight: *F* = 157.97; *df* = 3, 269; *p* < 0.001).

Group	Experimental Fish	*n*	Length (cm)	Weight (g)
small RC	Rock Carp (RC)	74	5.87 ± 0.09 ^d^	3.57 ± 0.15 ^d^
large RC	Rock Carp	75	9.04 ± 0.13 ^a^	13.53 ± 0.62 ^a^
small PS	Prenant’s Schizothoracin (PS)	71	6.38 ± 0.09 ^c^	4.32 ± 0.16 ^c^
large PS	Prenant’s Schizothoracin	73	8.09 ± 0.10 ^b^	8.23 ± 0.29 ^b^

**Table 2:** Relationship between the average survival time and the TDG supersaturation, which varied with species and size at the different supersaturation levels. Values are expressed the as mean ± SE, *n* = 12–15. Letters following the average survival time indicates the results from a post hoc multiple comparison test between groups at each supersaturation level. Mean values that do not share a common lowercase letter are significantly different (*p* < 0.05).

TDG	Group	Average Survival Time	df	F	*p*
145%	small RC	3.28 ± 0.09 ^a^	3.57	35.38	< 0.001
	large RC	2.23 ± 0.03 ^bc^			
	small PS	2.44 ± 0.14 ^b^			
	large PS	2.02 ± 0.08 ^c^			
140%	small RC	3.23 ± 0.05 ^a^	3.60	11.71	< 0.001
	large RC	2.68 ± 0.11 ^bc^			
	small PS	3.74 ± 0.17 ^b^			
	large PS	3.10 ± 0.15 ^c^			
135%	small RC	4.40 ± 0.11 ^a^	3.55	10.10	< 0.001
	large RC	5.24 ± 0.20 ^bc^			
	small PS	4.39 ± 0.46 ^b^			
	large PS	3.25 ± 0.16 ^c^			
130%	small RC	7.18 ± 0.82 ^a^	3.56	9.47	< 0.001
	large RC	6.03 ± 0.30 ^bc^			
	small PS	15.00 ± 1.87 ^b^			
	large PS	13.29 ± 1.81 ^c^			
125%	small RC	11.91 ± 1.03 ^a^	3.52	13.44	< 0.001
	large RC	6.53 ± 0.19 ^bc^			
	small PS	18.22 ± 2.49 ^b^			
	large PS	17.21 ± 1.93 ^c^			

**Table 3:** Catalase (CAT) activities of Rock Carp and Prenant’s Schizothoracin at different TDG levels. Values are expressed as the mean ± SE (*n* = 5–16). Letters following the CAT activities indicates the results from a post hoc multiple comparison test of small PS between different supersaturation levels. Mean values that do not share a common lowercase letter are significantly different (*p* < 0.05).

TDG	Group	CAT Activities	df	F	*p*
145%	large RC	34.82 ± 4.27	2, 34	8.8	0.001
	small PS	74.58 ± 7.15 ^a^			
	large PS	82.05 ± 8.69			
140%	large RC	32.38 ± 4.70	2, 27	9.97	0.001
	small PS	85.33 ± 13.36 ^a^			
	large PS	102.56 ± 9.41			
135%	large RC	38.52 ± 4.94	2, 31	16.34	< 0.001
	small PS	70.00 ± 8.09 ^a^			
	large PS	88.10 ± 6.18			
130%	large RC	47.33 ± 8.22	2, 38	8.7	0.001
	small PS	120.00 ± 14.73 ^b^			
	large PS	116.89 ± 11.04			
125%	large RC	35.56 ± 6.36	2, 20	6.54	0.007
	small PS	124.00 ± 29.18 ^ab^			
	large PS	87.22 ± 10.84			

**Table 4:** Comparison of CAT activity between treatment fish and control fish. Bold numbers indicate that the enzymatic activity of this group was higher than that of the control group.

TDG	Large RC	Small PS	Large PS
145%	34.82	74.58	82.05
140%	32.38	85.33	102.56
135%	38.52	70.00	88.10
130%	**47.33**	**120.00**	**116.89**
125%	35.56	**124.00**	87.22
100%	43.34	86.67	88.79
